# Metabolite Signature of Simvastatin Treatment Involves Multiple Metabolic Pathways

**DOI:** 10.3390/metabo12080753

**Published:** 2022-08-16

**Authors:** Lilian Fernandes Silva, Rowmika Ravi, Jagadish Vangipurapu, Markku Laakso

**Affiliations:** 1 Institute of Clinical Medicine, Internal Medicine, University of Eastern Finland, 70210 Kuopio, Finland; 2 Department of Medicine, Kuopio University Hospital, 70210 Kuopio, Finland

**Keywords:** simvastatin, metabolomics, metabolites

## Abstract

Statins inhibit the 3-hydroxy-3-methylglutaryl-CoA reductase enzyme and are the most widely used medication for hypercholesterolemia. Previous studies on the metabolite signature of simvastatin treatment have included only a small number of metabolites. We performed a high-throughput liquid chromatography–tandem mass spectroscopy profiling on the effects of simvastatin treatment on 1098 metabolite concentrations in the participants of the METSIM (Metabolic Syndrome In Men) study including 1332 participants with simvastatin treatment and 6200 participants without statin treatment. We found that simvastatin exerts profound pleiotropic effects on different metabolite pathways, affecting not only lipids, but also amino acids, peptides, nucleotides, carbohydrates, co-factors, vitamins, and xenobiotics. We identified 321 metabolites significantly associated with simvastatin treatment, and 313 of these metabolites were novel. Our study is the first comprehensive evaluation of the metabolic signature of simvastatin treatment in a large population-based study.

## 1. Introduction

Hyperlipidemia is recognized as a major risk factor for ischemic heart disease and coronary mortality [1,2], a leading cause of death worldwide. An estimated 7.2 million people die each year from coronary heart disease [3]. Multiple trials have shown that statins significantly decrease low-density-lipoprotein cholesterol (LDLC) concentration and the risk of cardiovascular diseases [4,5]. Statins inhibit the 3-hydroxy-3-methylglutaryl-CoA reductase (HMGCR) enzyme and are the most widely used medication for hypercholesterolemia [6]. However, statin therapy increases the risk of diabetes [7]. Two Mendelian randomization studies have assessed whether an increase in the risk of diabetes is a consequence of inhibition of HMGCR. They used genetic variants in the *HMGCR* gene as proxies for HMGCR inhibition by statins, and both studies confirmed a causal association between statin treatment and an increased risk of type 2 diabetes (T2D) [8,9].

Statins also have beneficial effects not related to dyslipidemia. Simvastatin increases endothelial function [10], blocks the platelet-derived growth factor and fibrinogen-induced smooth muscle proliferation and migration [11], reduces plasminogen activation inhibitor [12], stabilizes atheromatous plaques [13], prevents the inhibitive action exerted by oxidized LDL on nitric oxide [14], and is anti-inflammatory [15]. These ‘pleiotropic’ effects of statins are not fully understood.

Metabolomics is a powerful tool to investigate metabolite profiles [16,17]. The largest study published so far to identify biomarkers associated with simvastatin treatment applied a nuclear magnetic resonance (NMR) spectroscopy method and included only 80 metabolites [18]. Mass spectrometry is a highly sensitive and accurate method for detection and quantitation of metabolites in a single measurement [19]. We performed a high-throughput liquid chromatography–tandem mass spectroscopy profiling on the effects of simvastatin treatment on metabolite concentrations in our large Finnish population-based cohort, the Metabolic Syndrome In Men (METSIM) study, including 7532 participants. Our hypothesis is that investigating the metabolite signature of simvastatin treatment offers a novel approach of obtaining new information about metabolic pathways explaining ‘pleiotropic’ effects of statins.

## 2. Materials and Methods

### 2.1. Participants

The participants of our study were selected from the METSIM study comprising 10197 Finnish men randomly selected from the population register of Kuopio, Eastern Finland, aged from 45 to 73 years at baseline. The study design has been described previously in detail [20,21]. We excluded participants with diabetes defined by the American Diabetes Association criteria [22] from our analyses because hyperglycemia has effects on metabolite concentrations. Our study included 1332 participants on simvastatin treatment and 6200 participants without statin treatment. This subset of the METSIM study had similar clinical and laboratory characteristics as the entire METSIM population, and therefore it is a representative of the entire METSIM cohort. The study was approved by the Ethics Committee of the Kuopio University Hospital. All study participants gave written informed consent. All laboratory methods, including metabolomics analysis, were performed in accordance with the relevant guidelines and regulations.

### 2.2. Clinical and Laboratory Measurements

Height was measured without shoes to the nearest 0.5 cm. Weight was measured in light clothing with a calibrated digital scale (Seca 877, Hamburg, Germany), and rounded up to the nearest 0.1 kg. BMI was calculated as weight (kg) divided by height (m) squared. Waist and hip circumferences were measured to the nearest 0.5 cm. Laboratory studies after 12 h of fasting included plasma glucose and insulin, other relevant laboratory characteristics, and metabolomics (Metabolon, Durham, NC). An oral glucose tolerance test was performed to evaluate glucose tolerance (75 g of glucose). Clinical and laboratory measurement methods have been previously published [21]. Plasma glucose was measured by enzymatic hexokinase photometric assay (Konelab Systems Reagents, Thermo Fischer Scientific, Vantaa, Finland), and insulin by immunoassay (ADVIA Centaur Insulin IRI no. 02230141; Siemens Medical Solutions Diagnostics, Tarrytown, NY, USA). Total triglycerides, and low-density lipoprotein (LDL) cholesterol were measured using enzymatic colorimetric methods (Konelab Systems Reagents; Thermo Fisher Scientific, Vantaa, Finland).

### 2.3. Calculations

The Matsuda insulin sensitivity index (Matsuda ISI) and early-phase insulin secretion index (InsAUC_0–30_/GluAUC_0–30_) were calculated as previously described [21]. The disposition index (a marker of insulin secretion) was calculated as the Matsuda ISI × InsAUC_0–30_/GluAUC_0–30_.

### 2.4. Metabolomics Analysis

Metabolites were measured as part of Metabolon Inc.’s untargeted Discovery HD4 platform (Metabolon, Morrisville, NC, USA), as previously described in detail [23]. Briefly, methanol extraction of biochemicals followed by a non-targeted relative quantitative liquid chromatography–tandem mass spectrometry (LC-MS/MS) were performed. The Metabolon Discovery HD4 platform was applied to assay named and unnamed metabolites. A total of 1098 unique metabolites were included in statistical analysis. The classification of the metabolites was based on the Human Metabolome Database (http://www.hmdb.ca, accessed on 1 June 2022).

### 2.5. Statistical Analysis

All statistical analyses were performed using IBM SPSS Statistics 25. We logarithmically transformed all variables due to their skewed distributions. We used one-way ANOVA to assess the differences in clinical and laboratory traits, and metabolites between the participants on simvastatin treatment and the participants without statin treatment. Next, we performed ANCOVA analyses on age, BMI, fasting glucose, and smoking as confounding factors. Metabolites having a *p* value < 4.5 × 10^−5^ were considered statistically significant given the 1098 metabolites included in the analysis.

## 3. Results

Table 1 shows baseline characteristics of the participants included in our study. There were small but statistically significant differences in age, BMI, waist circumference, glucose and insulin concentrations, LDL cholesterol, insulin sensitivity index (Matsuda ISI), and insulin secretion index (Disposition index) between the participants on simvastatin treatment and the participants without statin treatment.

**Figure 1:**
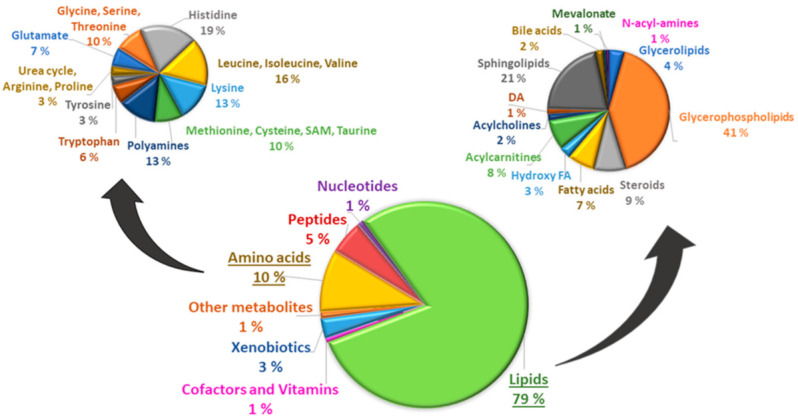
Lipids (79%) and amino acids (10%) were the most frequent groups of metabolites in 7532 participants included in our study. Among the lipids, glycerophospholipids (41%) were the most frequent subgroup of lipids, and histidine (19%) the most frequent subgroup of amino acids.

Figure 1 shows the main classes and subclasses of metabolites significantly associated with simvastatin treatment. Amongst a total of 321 metabolites significantly associated with simvastatin treatment 79% were lipids. Simvastatin treatment was also significantly associated with other metabolite groups including amino acids (10%), peptides (5%), xenobiotics (3%), and co-factors and vitamins (1%).

Supplementary Table S1 shows the individual metabolites significantly associated with simvastatin treatment compared to participants without statin treatment. We found 313 novel previously unpublished metabolite associations in participants on simvastatin treatment compared to the control group. The most significant novel association in the amino acid class was with betaine (*p* = 2.1 × 10^−48^), a metabolite belonging to the glycine pathway. In the peptide class the most significant association of simvastatin treatment was with gamma-glutamylglutamate (*p* = 6.0 × 10^−16^), in the nucleotides class with uridine (*p* = 1.9 × 10^−9^), in the carbohydrates pathway N-acetylglucosaminylasparagine (*p* = 2.2 × 10^−18^), in the lipids class cholesterol (*p* = 1.0 × 10^−196^) and glycosyl ceramide (d18:1/20:0, d16:1/22:0) (*p* = 5.6 × 10^−122^), and in the xenobiotics class 2-hydroxyhippurate (salicylurate) (*p* = 4.7 × 10^−38^). 

## 4. Discussion

Previous studies investigating the metabolic signature of simvastatin treatment have included only a small number of metabolites [18,24]. Our study shows for the first time a comprehensive metabolic signature of simvastatin treatment in 7532 participants with a total of 1098 metabolites measured.

Our study reports statistically significant changes in multiple metabolite concentrations in the participants on simvastatin treatment indicating that simvastatin exerts profound pleiotropic effects on different metabolite pathways, affecting not only lipids, but also amino acids, peptides, nucleotides, carbohydrates, co-factors, vitamins, and xenobiotics. We found 321 statistically significant differences in metabolite concentrations between the participants on simvastatin treatment compared to the participants not on statin treatment. A total of 313 of these associations were novel.

**Figure 2:**
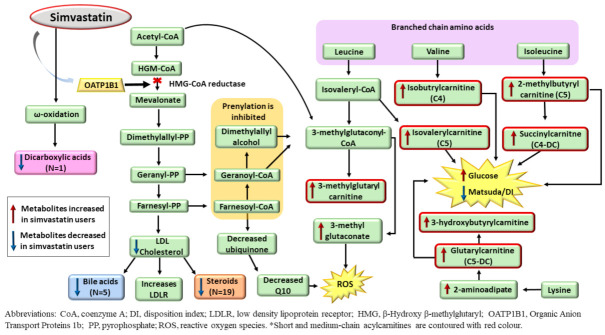
Effects of simvastatin treatment on steroids, bile acids, dicarboxylic acids, branched-chain amino acids, lysine and short-chain acylcarnitines.

Simvastatin is transported into the liver by OATP1B1 transporter where it inhibits the HMG-CoA reductase enzyme [25] that suppresses the synthesis of mevalonate, cholesterol, and its downstream metabolites (Figure 2). Simvastatin inhibits prenylation [26] and activates AMP-protein kinase (AMPK), leading to the accumulation of acetyl-CoA and generation of 3-methylglutaconate (3-MG) [27].

Simvastatin is transported into the liver by OATP1B1 transporter, where it inhibits HMG-CoA reductase, resulting in an increase in LDL receptor expression in the liver. Cholesterol synthesis is decreased, and consequently, downstream metabolites of cholesterol, such as bile acids and steroids, are decreased. Inhibition of HMG-CoA reductase results in an accumulation of acetyl-CoA, which can be directed to generate 3-methylglutaconate, leading to increased ROS generation. Simvastatin also leads to an increased metabolism of branched-chain amino acids and lysine, which generates an increase in short-chain acylcarnitines (C4, C4-DC, C5, and C5-DC).

As expected, the participants on simvastatin treatment had decreased concentrations of LDLC, bile acids, and steroids in our study. Although a decrease in total bile acids and testosterone in individuals on statin treatment has been previously described [28,29], we report for the first time that metabolites from these sub-pathways are associated with simvastatin treatment. We found 4 novel associations of simvastatin with secondary bile acids, and 18 novel associations with steroids, of which 7 belong to the androgenic steroid pathway and 8 to the pregnenolone pathway, suggesting that simvastatin regulates steroidogenesis. Additionally, we found higher concentrations of 3-MG in the participants on simvastatin treatment. Increased 3-MG is found in patients with statin-treatment-induced myopathy [30] and triggers oxidative stress in animal models [31].

Statins have been previously shown to upregulate mitochondrial acylcarnitine (AC) carrier gene expression [32], but a detailed metabolite profile of individual ACs has not been previously elucidated. We found novel increased concentrations of six novel short-chain ACs ((S)-3-hydroxybutyrylcarnitine, isobutyrylcarnitine (C4), isovalerylcarnitine (C5), 2-methylbutyrylcarnitine (C5), glutarylcarnitine (C5-DC), succinylcarnitine (C4-DC), and three medium-chain acylcarnitines, hexanoylcarnitine (C6), octanoylcarnitine (C8), cis-3,4-methyleneheptanoylcarnitine). Five of these metabolites were downstream metabolites of the degradation of the BCAAs (leucine, valine, isoleucine), and two were downstream metabolites of lysine degradation (Figure 2). Previous studies have reported that BCAA-derived C5-AC concentrations were higher in obese individuals and individuals with T2D compared with lean controls [32,33]. C4-DC AC, derived from BCAA metabolism, had a positive correlation with fasting glucose levels and HbA1c in a previous study [33]. There were increased concentrations of short-chain ACs in the participants on simvastatin treatment in our study; these participants also had increased concentrations of fasting and 2h glucose, and decreased insulin sensitivity and insulin secretion compared to participants not on statin treatment. Simvastatin has been shown to be associated with an increased risk of T2D [7,8,9,34], and short-chain ACs may increase this risk.

Although the lowering of lipid concentrations by simvastatin is well-known, the metabolic profile of lipids in individuals on statin treatment remains largely unknown given the lack of large population-based studies. We show for the first time a detailed metabolic profile of different lipids regulated by simvastatin treatment. Simvastatin downregulates two enzymes, fatty acid synthase (FAS) and diacylglycerol acyltransferase (DGAT) [35,36]. These enzymes decrease the synthesis of fatty acids (FAs) and the transfer of diacylglycerol (DAG) to neutral lipids, such as triacylglycerol. Simvastatin treatment also leads to upregulation of lipid phosphate phosphohydrolase 1, an enzyme that converts phosphatidic acid to DAG [37]. We found that the participants on simvastatin treatment had increased concentrations of eight DAGs, and decreased concentrations of two monoacylglycerols (MAGs) and eight long-chain FAs. Additionally, we found novel associations of simvastatin treatment with lower concentrations of 14 long-chain ACs, 15 plasmalogens, and 6 lyso-plasmalogens. Long-chain FAs are the precursors of fatty acyl-CoA, which is needed for the assembling of plasmalogens and its derivatives, lyso-plasmalogens, and for long-chain acylcarnitine generation [38,39]. Therefore, low concentrations of long-chain FAs might explain low concentrations of the metabolites having fatty acyl-CoA as their precursors.

**Figure 3:**
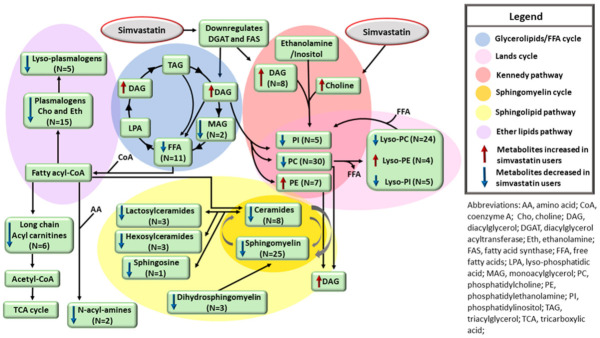
Effects of simvastatin treatment on lipid pathways.

Simvastatin increases choline levels [18], probably due to a decrease in the phosphocholine cytidylyltransferase activity, which prevents choline from being incorporated into phosphatidylcholine (PC) and sphingomyelins [40]. We confirmed increased concentrations of choline in the participants on simvastatin treatment and found new associations of simvastatin with decreased concentrations of 20 PCs and 24 lyso-PCs. Concentrations of ceramides, sphingomyelins, and the downstream metabolites hexosylceramides, sphingosines, and lactosylceramides were also decreased in the participants on simvastatin treatment. Decreased concentrations of sphingolipids could be explained by a decrease in fatty acyl-CoA coming from FAs. Additionally, it is possible that choline coming from PCs hydrolysis cannot be incorporated into the sphingosine backbone for sphingomyelin production [40,41,42]. Thus, simvastatin interferes with several pathways involved in lipid metabolism, including glycerolipids/free FA cycle, Lands cycle, sphingolipid pathway, and ether lipid pathway (Figure 3).

Simvastatin downregulates DGAT, which in turn decreases DAG incorporation into TAG droplets in the liver, resulting in an increase in DAG, and decreases in MAG and FFA concentrations. Simvastatin also decreases the incorporation of choline into PC and decreases the rate of the Kennedy pathway. Low concentrations of phospholipids (PCs and PIs) decrease the turnover of the Lands cycle and decrease the levels of lysophospholipids (lyso-PCs and lyso-PIs). PC is needed for sphingomyelin assembling, and low concentrations of PC decrease the concentrations of sphingomyelins, ceramides, and its downstream metabolites. FFAs are the precursors of long-chain acylcarnitines, N-acyl-amines, and the ether lipid pathway *via* generation of fatty acyl-CoA. Consequently, low levels of fatty acids result in decreased concentrations of long-chain acylcarnitines, N-acyl-amines, plasmalogens, and lyso-plasmalogens.

**Figure 4:**
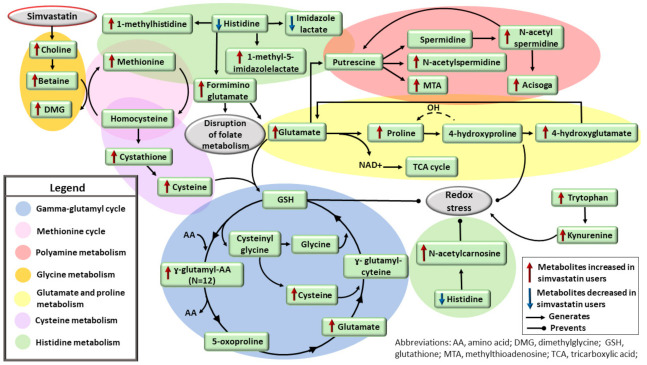
Effects of simvastatin treatment on amino acid and peptide pathways and redox balance.

We showed that simvastatin has important effects on amino acid pathways, in contrast to a previous study suggesting that simvastatin has minimal effects on amino acids [18] (Figure 4). Our novel findings are that simvastatin increased concentrations of betaine, dimethylglycine, methionine, cystathione, and cysteine. Choline can be oxidized to betaine [43]. Betaine donates a methyl group to homocysteine to form methionine, generating dimethylglycine [44]. Homocysteine can be converted to cystathione, which can further be converted to cysteine [45]. Cysteine enters the gamma-glutamyl cycle to generate glutathione, which is critical to the response to oxidative stress [46]. Glutathione is reduced to cysteinyl-glycine and gamma-glutamyl amino acids [47]. The metabolism of glutathione promotes the release and recovery of constituent amino acids, such as glutamate and cysteine. We found increased levels of 12 novel gamma-glutamyl amino acids and glutamate in the participants on simvastatin use, indicating disruption of the gamma-glutamyl cycle in these participants.

Simvastatin increases levels of choline, which is oxidized to betaine. Betaine donates a methyl group to homocysteine to form methionine, generating dimethylglycine. Homocysteine can be converted to cystathione, and further to cysteine, which enters the ɣ-glutamyl cycle to generate GSH. GSH is critical to the response to oxidative stress. GSH is reduced by ɣ-glutamyl transferase (GGT) to cysteinyl-glycine and ɣ-glutamyl amino acids. Metabolism of GSH by GGT promotes the release and recovery of constituent amino acids, such as glutamate and cysteine. Increased levels of ɣ-glutamyl amino acids and glutamate indicates disruption of the ɣ-glutamyl cycle. Simvastatin decreases histidine levels and its downstream metabolite imidazole lactate, and increases levels of 1-methylhistidine, 1-methyl-5-imidazolelactate, formiminoglutamate, and N-acetylcarnosine. N-acetylcarnosine is a free-radical scavenger. Elevated levels of formiminoglutamate indicate disruption of folate metabolism. Formiminoglutamate can be converted to glutamate, which can be used to synthetize proline. Proline synthesis from glutamate helps to regenerate NAD+ towards the TCA cycle and facilitate ROS scavenging. Glutamate can generate putrescine via a series of enzymatic reactions, impacting the polyamine metabolism. Tryptophan and kynurenine were increased in the participants on simvastatin treatment. Kynurenine increases oxidative stress.

In our study, the participants on simvastatin treatment had decreased concentrations of histidine, as previously reported [18], and decreased concentrations of its downstream metabolite imidazole lactate. We found increased concentrations of four novel metabolites in the histidine pathway, 1-methylhistidine, 1-methyl-5-imidazolelactate formiminoglutamate, and N-acetylcarnosine. N-acetylcarnosine is a free-radical scavenger and is particularly active against lipid peroxidation [48]. Elevated concentrations of formiminoglutamate indicate disruption of folate metabolism [49]. Formiminotransferase converts formiminoglutamate to glutamate [45], and glutamate can be used to synthetize proline [45]. Simvastatin increases proline concentrations in mice [50]. Proline itself can act as a reactive oxygen species (ROS) scavenger [51,52]. Thus, proline synthesis from glutamate helps to regenerate NAD+ towards the tricarboxylic acid cycle and facilitate ROS scavenging (Figure 4). Glutamate can generate putrescine through a series of enzymatic reactions [45]. We found increased levels of four metabolites belonging to the polyamine pathway. Tryptophan and kynurenine were also increased in the participants on simvastatin treatment. Kynurenine has been shown to increase oxidative stress [53].

In conclusion, the metabolic signature of simvastatin treatment is more complex than previously thought. Our novel findings were that the metabolic signature of simvastatin involves disturbances in several metabolite pathways, including lipids, steroids, degradation of BCAA, disturbances in the gamma-glutamyl cycle, folate, glutamate, and proline metabolism. Our findings show that the metabolic signature of simvastatin treatment includes metabolites involved in the generation of ROS, but also metabolites that act as ROS scavengers to keep the redox balance. Mechanistic studies are needed to investigate the role of these metabolites in simvastatin treatment. Therefore, it is too early to predict the clinical importance of multiple metabolic pathways we identified in the pleiotropic effects of statins.

The strength of our study is the large size of our population-based study, detailed metabolite analyses, and identification of several novel metabolites (especially short-chain acyl-carnitines and amino acids) associated with simvastatin treatment. The limitation of our study is that only middle-aged and elderly Finnish men were included, and therefore we do not know if the results are valid for women, all age groups, and other ethnic and racial groups.

## Figures and Tables

**Table 1:** Clinical and laboratory characteristics of participants without statin treatment and participants on simvastatin treatment.

Clinical and Laboratory Characteristics	Participants on Simvastatin Treatment *			Participants Not on Simvastatin Treatment *			*p* Value **
	*n*	Mean	SD	*n*	Mean	SD	
Age	1332	59.74	7,08	6200	56.62	6.92	<0.001
Body mass index	1331	27.43	3.96	6198	26.60	3.74	<0.001
Waist (cm)	1331	99.11	10.85	6197	96.84	10.55	<0.001
Systolic blood pressure	1332	137.88	15.82	6200	136.81	16.28	NS
Fasting plasma glucose (mmol/L)	1332	6.42	1.74	6200	5.93	1.65	<0.001
2 h plasma glucose (mmol/L)	1332	5.79	0.48	6200	5.69	0.48	<0.001
Fasting plasma insulin (mU/L)	1331	9.53	6.93	6197	7.82	5.52	<0.001
Matsuda ISI (mg/dl, mU/L)	1320	5.71	3.44	6167	7.33	4.29	<0.001
Disposition index	1320	153.22	65.16	6167	166.90	73.62	<0.001
LDL cholesterol (mmol/L)	1332	2.71	0.71	6197	3.57	0.82	<0.001
Total triglycerides (mmol/L)	1332	1.41	0.72	6200	1.38	1.00	NS
Smokers %		13.0%			16.6%	-	NS

* Only participants with metabolites available were included in statistical analyses. ** *p* value for ANOVA. Abbreviations: Matsuda ISI, Matsuda insulin sensitivity index; disposition index, insulin secretion index; NS, not statistically significant.

## Data Availability

The data presented in this study are available on request from the corresponding author. The data are not publicly available due to preserving the confidentiality of the participants.

## Supplementary Materials

The following supporting information can be downloaded at: https://www.mdpi.com/article/10.3390/metabo12080753/s1, Table S1: Comparison between the levels of the metabolites in participants on simvastatin treatment and in participants not on simvastatin treatment.
