# Supplementary material for: Metabolite Signature of Simvastatin Treatment Involves Multiple Metabolic Pathways
Source: Metabolites. 2022 Aug 16;12(8):753. doi: 10.3390/metabo12080753 (PMC9414498; doi:10.3390/metabo12080753)
Supplement: Supplementary file 1 [file metabolites-12-00753-s001.zip › metabolites-1859853-supplementary.pdf]

Table S1. Comparison between the levels of the metabolites in participants on simvastatin treatment and in participants not on simvastatin treatment.

Metabolite	HMDB	Sub-class	Participants on simvastatin treatment			Participants not on simvastatin treatment					
			N	Mean	SE	N	Mean	SE	P-value	P-value*	Novel
**Amino acids**											
Glutamate	HMDB0000148	Glutamate Metabolism	1332	0,168	0,026	6200	-0,056	0,013	1,23E-13	6,64E-09	YES
**4-hydroxyglutamate**	**HMDB0001344**	**Glutamate Metabolism**	**1026**	**0,231**	**0,031**	**4449**	**-0,081**	**0,015**	**0,00E+00**	**4,42E-14**	YES
Threonine	HMDB0000167	Glycine, Serine and Threonine Metabolism	1332	0,141	0,027	6200	-0,045	0,013	7,14E-10	2,87E-12	YES
**Betaine**	**HMDB0000043**	**Glycine, Serine and Threonine Metabolism**	**1331**	**0,314**	**0,026**	**6198**	**-0,105**	**0,013**	**0,00E+00**	**0,00E+00**	YES
**Dimethylglycine**	**HMDB0000092**	**Glycine, Serine and Threonine Metabolism**	**1332**	**0,266**	**0,028**	**6197**	**-0,072**	**0,013**	**0,00E+00**	**0,00E+00**	YES
Histidine	HMDB0000177	Histidine Metabolism	1332	-0,162	0,026	6200	0,047	0,013	4,80E-12	1,67E-07	No(31)
Imidazole lactate	HMDB0002320	Histidine Metabolism	1332	-0,118	0,027	6192	0,034	0,013	4,03E-07	4,97E-08	YES
1-methylhistidine	HMDB0000001	Histidine Metabolism	1327	0,168	0,03	6188	-0,052	0,012	2,16E-13	4,75E-08	YES
**Formiminoglutamate**	**HMDB0000854**	**Histidine Metabolism**	**1101**	**0,285**	**0,029**	**4352**	**-0,107**	**0,015**	**0,00E+00**	**0,00E+00**	YES
N-acetylcarnosine	HMDB0012881	Histidine Metabolism	1330	0,098	0,027	6179	-0,033	0,013	1,42E-05	1,81E-06	YES
1-methyl-5-imidazolelactate	-	Histidine Metabolism	1267	0,118	0,028	5885	-0,044	0,013	1,52E-07	5,74E-06	YES
Isobutyrylcarnitine (C4)	HMDB0000736	Leucine, Isoleucine and Valine Metabolism	1330	0,14	0,028	6184	-0,052	0,013	1,69E-10	6,55E-08	YES
Isovalerylcarnitine (C5)	HMDB0000688	Leucine Metabolism	1331	0,138	0,028	6200	-0,052	0,013	2,43E-10	3,48E-07	YES
2-methylbutyrylcarnitine (C5)	HMDB0000378	Leucine, Isoleucine and Valine Metabolism	1171	0,208	0,03	5277	-0,07	0,013	0,00E+00	4,81E-12	YES
**3-methylglutaconate**	**HMDB0000522**	**Leucine, Isoleucine and Valine Metabolism**	**1332**	**0,709**	**0,029**	**6196**	**-0,238**	**0,011**	**0,00E+00**	**0,00E+00**	**No(42)**
3-methylglutarylcarnitine (2)	HMDB0000552	Leucine, Isoleucine and Valine Metabolism	1325	0,553	0,028	6121	-0,191	0,012	0,00E+00	0,00E+00	YES
**2-aminoadipate**	**HMDB0000510**	**Lysine Metabolism**	**1145**	**0,29**	**0,029**	**5329**	**-0,099**	**0,013**	**0,00E+00**	**0,00E+00**	**No(30)**
Pipecolate	HMDB0000716	Lysine Metabolism	1327	-0,121	0,025	6181	0,039	0,013	1,39E-07	2,48E-07	YES
Glutarylcarnitine (C5-DC)	HMDB0013130	Lysine Metabolism	1332	0,109	0,028	6200	-0,045	0,013	3,17E-07	1,25E-05	YES
N,N,N-trimethyl-5-aminovalerate	-	Lysine Metabolism	1332	0,129	0,028	6200	-0,045	0,012	5,64E-09	6,02E-07	YES

**Cystathionine**	**HMDB0000099**	**Methionine, Cysteine, SAM and Taurine Metabolism**	**1191**	**0,226**	**0,031**	**5464**	**-0,072**	**0,013**	**0,00E+00**	**2,70E-10**	YES
Methionine	HMDB0000696	Methionine, Cysteine, SAM and Taurine Metabolism	1332	0,113	0,027	6200	-0,037	0,013	6,10E-07	1,04E-06	YES
Cysteine	HMDB0000574	Methionine, Cysteine, SAM and Taurine Metabolism	1332	0,111	0,026	6200	-0,043	0,013	3,47E-07	2,13E-05	YES
**N-acetylputrescine**	**HMDB0002064**	**Polyamine Metabolism**	**1331**	**0,16**	**0,027**	**6193**	**-0,054**	**0,013**	**1,29E-12**	**3,81E-08**	YES
5-methylthioadenosine (MTA)	HMDB0001173	Polyamine Metabolism	1330	0,202	0,026	6193	-0,069	0,013	0,00E+00	1,25E-07	YES
Acisoga	HMDB0061384	Polyamine Metabolism	1331	0,156	0,027	6114	-0,046	0,013	1,97E-11	2,26E-06	YES
(N(1) + N(8))-acetylspermidine	HMDB0002189	Polyamine Metabolism	1329	0,135	0,028	6185	-0,033	0,013	2,00E-08	4,14E-05	YES
Tryptophan	HMDB0000929	Tryptophan Metabolism	1332	0,116	0,026	6200	-0,033	0,013	8,14E-07	6,13E-06	YES
**Kynurenine**	**HMDB0000684**	**Tryptophan Metabolism**	**1332**	**0,226**	**0,027**	**6200**	**-0,066**	**0,013**	**0,00E+00**	**6,12E-09**	YES
3-methoxytyramine sulfate	-	Tyrosine Metabolism	783	0,152	0,036	2616	-0,052	0,019	4,97E-07	2,94E-05	YES
Proline	HMDB0000162	Urea cycle; Arginine and Proline Metabolism	1332	0,111	0,026	6199	-0,044	0,013	3,14E-07	8,01E-06	No(70)
**Peptides**											
**Phenylacetylcarnitine**	**-**	**Acetylated Peptides**	**1078**	**0,208**	**0,031**	**4556**	**-0,074**	**0,015**	**3,00E-17**	**6,50E-12**	YES
Phenylacetylglutamate	HMDB0059772	Acetylated Peptides	1017	0,179	0,033	4068	-0,065	0,015	1,82E-12	4,47E-07	YES
Cyclo(leu-pro)	HMDB0034276	Dipeptide	1199	0,166	0,028	5319	-0,065	0,014	4,88E-13	5,82E-07	YES
Phenylalanyltryptophan	HMDB0029006	Dipeptide	1282	-0,107	0,032	5918	0,022	0,012	2,09E-05	1,63E-06	YES
Valylglycine	HMDB0029127	Dipeptide	733	-0,17	0,038	3025	0,043	0,018	2,15E-07	1,49E-05	YES
Gamma-glutamyltyrosine	HMDB0011741	Ɣ-glutamyl Amino Acid	1328	0,189	0,026	6197	-0,057	0,013	5,70E-16	2,14E-06	YES
**Gamma-glutamylglutamate**	**HMDB0011737**	**Ɣ-glutamyl Amino Acid**	**1316**	**0,239**	**0,027**	**6062**	**-0,063**	**0,013**	**0,00E+00**	**6,00E-16**	YES
Gamma-glutamylleucine	HMDB0011171	Ɣ-glutamyl Amino Acid	1331	0,164	0,027	6190	-0,049	0,013	1,91E-12	8,58E-08	YES
Gamma-glutamylphenylalanine	HMDB0000594	γ-glutamyl Amino Acid	1332	0,201	0,028	6198	-0,063	0,013	0,00E+00	3,79E-07	YES
Gamma-glutamyl-epsilon-lysine	HMDB0003869	Ɣ-glutamyl Amino Acid	1063	0,18	0,032	5016	-0,056	0,014	1,82E-12	7,65E-11	YES
Gamma-glutamyltryptophan	HMDB0029160	Ɣ-glutamyl Amino Acid	1173	0,169	0,028	4913	-0,052	0,014	1,26E-11	8,85E-07	YES
Gamma-glutamylmethionine	HMDB0029155	Ɣ-glutamyl Amino Acid	1332	0,181	0,028	6199	-0,052	0,013	1,22E-14	2,68E-11	YES
Gamma-glutamylthreonine	HMDB0029159	Ɣ-glutamyl Amino Acid	1327	0,176	0,027	6142	-0,053	0,013	4,32E-14	1,62E-11	YES

Gamma-glutamylisoleucine	HMDB0011170	Ɣ-glutamyl Amino Acid	1332	0,155	0,028	6191	-0,047	0,013	2,68E-11	8,82E-06	YES
Gamma-glutamyl-2-aminobutyrate	HMDB0242176	Ɣ-glutamyl Amino Acid	1291	0,136	0,028	5999	-0,038	0,013	1,43E-08	7,10E-06	YES
Gamma-glutamylalanine	HMDB0029142	Ɣ-glutamyl Amino Acid	1060	0,138	0,032	5076	-0,04	0,014	1,55E-07	2,14E-05	YES
Gamma-glutamyl-alpha-lysine	-	Ɣ-glutamyl Amino Acid	1331	0,146	0,028	6190	-0,042	0,013	3,98E-10	2,88E-07	YES
**Nucleotides**											
Xanthine	HMDB0000292	Purine Metabolism, (Hypo)Xanthine/Inosine containing	1332	0,132	0,026	6198	-0,057	0,012	1,17E-10	1,07E-05	YES
Urate	HMDB0000289	Purine Metabolism, (Hypo)Xanthine/Inosine containing	1332	0,17	0,027	6200	-0,045	0,013	1,07E-12	1,35E-06	YES
**Uridine**	**HMDB0000296**	**Pyrimidine Metabolism, Uracil containing**	**1332**	**0,131**	**0,026**	**6200**	**-0,035**	**0,013**	**3,95E-08**	**1,92E-09**	YES
**Carbohydrates**											
**N-acetylglucosaminylasparagine**	**HMDB0000489**	**Aminosugar Metabolism**	**1091**	**-0,165**	**0,031**	**5153**	**0,068**	**0,014**	**1,30E-12**	**0,00E+00**	YES
Arabitol/xylitol	HMDB0001851	Pentose Metabolism	1331	0,133	0,028	6196	-0,046	0,013	3,06E-09	4,23E-05	YES
**Lipids**											
**Glycerolipids**											
Linoleoyl-arachidonoyl-glycerol (18:2/20:4) [1]*	HMDB0007257	Diacylglycerol	1216	0,227	0,028	5326	-0,093	0,014	0,00E+00	0,00E+00	YES
**Linoleoyl-arachidonoyl-glycerol (18:2/20:4) [2]***	**HMDB0007257**	**Diacylglycerol**	**1301**	**0,275**	**0,025**	**5822**	**-0,117**	**0,013**	**0,00E+00**	**0,00E+00**	YES
Linoleoyl-docosahexaenoyl-glycerol (18:2/22:6) [1]*	HMDB0007266	Diacylglycerol	1006	0,186	0,03	4290	-0,075	0,015	8,21E-14	5,92E-10	YES
Linoleoyl-docosahexaenoyl-glycerol (18:2/22:6) [2]*	HMDB0007266	Diacylglycerol	1155	0,187	0,027	4963	-0,073	0,014	1,54E-15	2,74E-10	YES
Oleoyl-arachidonoyl-glycerol (18:1/20:4) [1]*	HMDB0007228	Diacylglycerol	1300	0,271	0,026	5927	-0,115	0,013	0,00E+00	0,00E+00	YES
Oleoyl-arachidonoyl-glycerol (18:1/20:4) [2]*	HMDB0007228	Diacylglycerol	1267	0,247	0,026	5608	-0,108	0,013	0,00E+00	0,00E+00	YES
Linoleoyl-linoleoyl-glycerol (18:2/18:2) [1]*	HMDB0007248	Diacylglycerol	1248	0,139	0,027	5730	-0,051	0,013	8,33E-10	2,21E-08	YES
Stearoyl-arachidonoyl-glycerol (18:0/20:4) [1]*	HMDB0007170	Diacylglycerol	751	0,221	0,038	2799	-0,117	0,018	3,00E-17	0,00E+00	YES

2-linoleoylglycerol (18:2)	HMDB0011538	Monoacylglycerol	1276	-0,149	0,029	5876	0,052	0,013	7,05E-11	2,25E-09	YES
1-myristoylglycerol (14:0)	HMDB0011561	Monoacylglycerol	1227	-0,118	0,028	5815	0,026	0,013	4,43E-06	1,15E-07	YES
**Glycerophospholipids**											
Choline	HMDB0000097	Phospholipid Metabolism	1332	0,203	0,027	6199	-0,073	0,013	0,00E+00	1,65E-13	No(31)
Glycerophosphorylcholine (GPC)	HMDB0000086	Phospholipid Metabolism	1332	-0,252	0,028	6200	0,084	0,013	0,00E+00	0,00E+00	YES
Glycerophosphoethanolamine	HMDB0000114	Phospholipid Metabolism	1332	-0,13	0,028	6200	0,039	0,013	2,11E-08	2,62E-05	YES
**1-palmitoyl-2-linoleoyl-GPC (16:0/18:2)**	**HMDB0007973**	**Phosphatidylcholine**	**1331**	**-0,51**	**0,027**	**6190**	**0,179**	**0,012**	**0,00E+00**	**0,00E+00**	YES
1-palmitoyl-2-oleoyl-GPC (16:0/18:1)	HMDB0007972	Phosphatidylcholine	1331	-0,216	0,028	6189	0,061	0,012	0,00E+00	0,00E+00	YES
1,2-dipalmitoyl-GPC (16:0/16:0)	HMDB0000564	Phosphatidylcholine	1331	-0,362	0,024	6190	0,124	0,013	0,00E+00	0,00E+00	YES
1,2-distearoyl-GPC (18:0/18:0)	HMDB0008036	Phosphatidylcholine	207	-0,262	0,074	1584	0,055	0,025	1,48E-05	2,47E-05	YES
1-myristoyl-2-palmitoyl-GPC (14:0/16:0)	HMDB0007869	Phosphatidylcholine	1331	-0,307	0,027	6188	0,098	0,012	0,00E+00	0,00E+00	YES
1-stearoyl-2-arachidonoyl-GPC (18:0/20:4)	HMDB0008048	Phosphatidylcholine	1332	0,288	0,025	6194	-0,106	0,013	0,00E+00	0,00E+00	YES
1,2-dilinoleoyl-GPC (18:2/18:2)	HMDB0008138	Phosphatidylcholine	1322	-0,485	0,027	6142	0,162	0,012	0,00E+00	0,00E+00	YES
1-stearoyl-2-oleoyl-GPC (18:0/18:1)	HMDB0008038	Phosphatidylcholine	1331	-0,283	0,027	6179	0,074	0,012	0,00E+00	0,00E+00	YES
1-palmitoyl-2-arachidonoyl-GPC (16:0/20:4n6)	HMDB0007982	Phosphatidylcholine	1332	0,216	0,025	6196	-0,08	0,013	0,00E+00	0,00E+00	YES
1-palmitoyl-2-stearoyl-GPC (16:0/18:0)	HMDB0007970	Phosphatidylcholine	1330	-0,412	0,026	6176	0,134	0,012	0,00E+00	0,00E+00	YES
1-stearoyl-2-linoleoyl-GPC (18:0/18:2)*	HMDB0008039	Phosphatidylcholine	1332	-0,384	0,023	6191	0,128	0,013	0,00E+00	0,00E+00	YES
1-oleoyl-2-linoleoyl-GPC (18:1/18:2)*	HMDB0008105	Phosphatidylcholine	229	-0,383	0,069	1811	0,076	0,023	4,24E-11	3,83E-09	YES
1-palmitoyl-2-palmitoleoyl-GPC (16:0/16:1)*	HMDB0007969	Phosphatidylcholine	1332	-0,175	0,026	6197	0,05	0,013	7,28E-14	0,00E+00	YES
1-palmitoyl-2-eicosapentaenoyl-GPC (16:0/20:5)*	HMDB0007984	Phosphatidylcholine	1331	0,139	0,023	6187	-0,059	0,013	6,84E-11	8,79E-08	YES
1-pentadecanoyl-2-linoleoyl-GPC (15:0/18:2)*	HMDB0007940	Phosphatidylcholine	1331	-0,485	0,026	6186	0,181	0,012	0,00E+00	4,24E-09	YES
1-margaroyl-2-oleoyl-GPC (17:0/18:1)*	-	Phosphatidylcholine	1331	-0,148	0,029	6176	0,042	0,012	2,18E-10	8,61E-11	YES

1-margaroyl-2-linoleoyl-GPC (17:0/18:2)*	-	Phosphatidylcholine	1331	-0,306	0,027	6191	0,108	0,012	0,00E+00	0,00E+00	YES
1-palmitoleoyl-2-linoleoyl-GPC (16:1/18:2)*	HMDB0008006	Phosphatidylcholine	227	-0,346	0,067	1793	0,078	0,023	1,52E-09	3,92E-10	YES
1-pentadecanoyl-2-arachidonoyl-GPC (15:0/20:4)*	HMDB0007949	Phosphatidylcholine	1311	0,114	0,027	6086	-0,032	0,013	1,54E-06	5,36E-06	YES
1-pentadecanoyl-2-docosahexaenoyl-GPC (15:0/22:6)*	HMDB0007958	Phosphatidylcholine	1324	-0,111	0,025	6123	0,045	0,013	2,75E-07	5,43E-10	YES
1-oleoyl-2-docosahexaenoyl-GPC (18:1/22:6)*	HMDB0008123	Phosphatidylcholine	1331	0,101	0,025	6184	-0,049	0,013	5,97E-07	7,75E-07	YES
1-myristoyl-2-linoleoyl-GPC (14:0/18:2)*	HMDB0007874	Phosphatidylcholine	1331	-0,423	0,025	6181	0,143	0,012	0,00E+00	0,00E+00	YES
1-myristoyl-2-docosahexaenoyl-GPC (14:0/22:6)*	HMDB0007892	Phosphatidylcholine	1301	-0,137	0,025	6054	0,038	0,013	9,83E-09	1,21E-10	YES
1-stearoyl-2-docosapentaenoyl-GPC (18:0/22:5n3)*	HMDB0008056	Phosphatidylcholine	1332	-0,127	0,026	6192	0,021	0,013	8,69E-07	3,81E-07	YES
1-palmitoyl-2-adrenoyl-GPC (16:0/22:4)*	HMDB0007988	Phosphatidylcholine	1330	-0,271	0,028	6177	0,076	0,012	0,00E+00	0,00E+00	YES
1-stearoyl-2-adrenoyl-GPC (18:0/22:4)*	HMDB0008054	Phosphatidylcholine	1331	-0,199	0,028	6179	0,048	0,012	1,20E-16	1,00E-17	YES
Phosphatidylcholine (18:0/20:5, 16:0/22:5n6)*	-	Phosphatidylcholine	1322	0,194	0,025	6081	-0,084	0,013	0,00E+00	1,41E-15	YES
1-linoleoyl-2-linolenoyl-GPC (18:2/18:3)*	HMDB0008141	Phosphatidylcholine	1314	-0,431	0,028	6129	0,141	0,012	0,00E+00	0,00E+00	YES
1-palmitoleoyl-2-linolenoyl-GPC (16:1/18:3)*	HMDB0008008	Phosphatidylcholine	1208	-0,338	0,027	5608	0,107	0,013	0,00E+00	0,00E+00	YES
Phosphatidylcholine (14:0/14:0, 16:0/12:0)	-	Phosphatidylcholine	1282	-0,383	0,027	5929	0,119	0,013	0,00E+00	0,00E+00	YES
Phosphatidylcholine (15:0/18:1, 17:0/16:1, 16:0/17:1)*	-	Phosphatidylcholine	1331	-0,289	1331	6179	0,095	0,012	0,00E+00	0,00E+00	YES
1-oleoyl-2-docosapentaenoyl-GPC (18:1/22:5n3)*	HMDB0008122	Phosphatidylcholine	1299	-0,166	0,028	6087	0,043	0,013	8,71E-12	7,58E-08	YES
Phosphatidylcholine (18:0/20:2, 20:0/18:2)*	-	Phosphatidylcholine	1269	-0,313	0,026	5775	0,092	0,013	0,00E+00	0,00E+00	YES

1-linoleoyl-2-docosapentaenyol-GPC (18:2/22:5n3)*	-	Phosphatidylcholine	845	-0,35	0,034	4595	0,093	0,014	0,00E+00	0,00E+00	YES
1-palmitoyl-2-pentadecanoyl-GPC (16:0/15:0)*	HMDB0007967	Phosphatidylcholine	1330	-0,344	0,027	6177	0,124	0,012	0,00E+00	0,00E+00	YES
1-palmitoyl-2-alpha-linolenoyl-GPC (16:0/18:3n3)*	-	Phosphatidylcholine	332	-0,482	0,059	2420	0,119	0,019	0,00E+00	0,00E+00	YES
1-palmitoyl-2-oleoyl-GPE (16:0/18:1)	HMDB0005320	PE	1324	0,112	0,026	6113	-0,057	0,013	2,21E-08	1,95E-06	YES
1-palmitoyl-2-linoleoyl-GPE (16:0/18:2)	HMDB0005322	PE	1329	-0,186	0,026	6170	0,054	0,013	2,07E-15	8,92E-15	YES
1-stearoyl-2-linoleoyl-GPE (18:0/18:2)*	HMDB0008994	PE	1327	-0,127	0,025	6150	0,031	0,013	1,75E-07	2,43E-07	YES
1-stearoyl-2-arachidonoyl-GPE (18:0/20:4)	HMDB0009003	PE	1329	0,228	0,026	6172	-0,085	0,013	0,00E+00	0,00E+00	YES
1-palmitoyl-2-arachidonoyl-GPE (16:0/20:4)*	HMDB0005323	PE	1330	0,111	0,027	6156	-0,05	0,013	9,44E-08	4,09E-08	YES
1-palmitoyl-2-docosahexaenoyl-GPE (16:0/22:6)*	HMDB0008946	PE	1329	0,275	0,025	6174	-0,103	0,013	0,00E+00	0,00E+00	YES
**1-stearoyl-2-docosahexaenoyl-GPE (18:0/22:6)***	**HMDB0009012**	**PE**	**1326**	**0,364**	**0,024**	**6140**	**-0,129**	**0,013**	**0,00E+00**	**0,00E+00**	YES
1-oleoyl-2-arachidonoyl-GPE (18:1/20:4)*	HMDB0009069	PE	1152	0,26	0,028	4624	-0,127	0,015	0,00E+00	0,00E+00	YES
1-oleoyl-2-docosahexaenoyl-GPE (18:1/22:6)*	HMDB0009078	PE	1091	0,349	0,029	4288	-0,164	0,015	0,00E+00	0,00E+00	YES
1-palmitoyl-2-linoleoyl-GPI (16:0/18:2)	HMDB0009784	Phosphatidylinositol	1279	-0,495	0,027	5992	0,164	0,012	0,00E+00	0,00E+00	YES
1-stearoyl-2-arachidonoyl-GPI (18:0/20:4)	HMDB0009815	Phosphatidylinositol	1332	0,131	0,026	6199	-0,047	0,013	2,72E-09	7,86E-07	YES
1-palmitoyl-2-arachidonoyl-GPI (16:0/20:4)*	HMDB0009789	Phosphatidylinositol	1280	-0,207	0,028	5853	0,062	0,013	0,00E+00	0,00E+00	YES
1-stearoyl-2-linoleoyl-GPI (18:0/18:2)	HMDB0009809	Phosphatidylinositol	1331	-0,444	0,027	6194	0,149	0,012	0,00E+00	0,00E+00	YES
1-palmitoyl-2-oleoyl-GPI (16:0/18:1)*	HMDB0009783	Phosphatidylinositol	1302	-0,46	0,026	6098	0,141	0,012	0,00E+00	0,00E+00	YES
**1-stearoyl-2-oleoyl-GPI (18:0/18:1)***	**HMDB0240667**	**Phosphatidylinositol**	**1069**	**-0,573**	**0,031**	**4319**	**0,196**	**0,014**	**0,00E+00**	**0,00E+00**	YES
1-stearoyl-GPI (18:0)	HMDB0240261	Lyso-PC	1332	-0,221	0,026	6200	0,073	0,013	0,00E+00	0,00E+00	YES
1-palmitoyl-GPC (16:0)	HMDB0010382	Lyso-PC	1332	-0,314	0,026	6199	0,093	0,013	0,00E+00	0,00E+00	YES

1-margaroyl-GPC (17:0)	HMDB0012108	Lyso-PC	1332	-0,183	0,026	6200	0,056	0,013	1,63E-15	6,22E-11	YES
1-stearoyl-GPC (18:0)	HMDB0010384	Lyso-PC	1332	-0,331	0,027	6196	0,097	0,012	0,00E+00	0,00E+00	YES
1-oleoyl-GPC (18:1)	HMDB0002815	Lyso-PC	1332	-0,241	0,027	6199	0,065	0,013	0,00E+00	9,66E-15	YES
1-myristoyl-GPC (14:0)	HMDB0010379	Lyso-PC	1332	-0,246	0,025	6197	0,071	0,013	0,00E+00	0,00E+00	YES
**1-arachidoyl-GPC (20:0)**	**HMDB0010390**	**Lyso-PC**	**1316**	**-0,384**	**0,027**	**6105**	**0,121**	**0,013**	**0,00E+00**	**0,00E+00**	YES
1-linoleoyl-GPC (18:2)	HMDB0010386	Lyso-PC	1332	-0,38	0,027	6199	0,127	0,012	0,00E+00	0,00E+00	YES
1-stearoyl-GPE (18:0)	HMDB0011130	Lyso-PC	1331	-0,151	0,026	6177	0,034	0,013	8,45E-10	1,36E-06	YES
1-palmitoleoyl-GPC (16:1)*	HMDB0010383	Lyso-PC	1332	-0,15	0,026	6196	0,036	0,013	6,78E-10	1,51E-09	YES
1-arachidonoyl-GPC (20:4n6)*	HMDB0010395	Lyso-PC	1332	0,175	0,026	6198	-0,069	0,013	3,20E-16	0,00E+00	YES
1-dihomo-linoleoyl-GPC (20:2)*	HMDB0010392	Lyso-PC	1332	-0,362	0,027	6191	0,111	0,012	0,00E+00	0,00E+00	YES
2-arachidonoyl-GPC (20:4)*	HMDB0061699	Lyso-PC	1307	0,195	0,026	5911	-0,074	0,013	0,00E+00	0,00E+00	YES
2-oleoyl-GPC (18:1)*	HMDB0061701	Lyso-PC	1332	-0,222	0,028	6199	0,052	0,013	0,00E+00	2,68E-13	YES
2-linoleoyl-GPC (18:2)*	HMDB0061700	Lyso-PC	1326	-0,354	0,027	6164	0,116	0,012	0,00E+00	0,00E+00	YES
2-palmitoyl-GPC (16:0)*	HMDB0061702	Lyso-PC	1332	-0,24	0,028	6200	0,065	0,013	0,00E+00	0,00E+00	YES
2-myristoyl-GPC (14:0)*	HMDB0010379	Lyso-PC	1316	-0,24	0,026	6047	0,064	0,013	0,00E+00	0,00E+00	YES
1-pentadecanoyl-GPC (15:0)*	HMDB0010381	Lyso-PC	1332	-0,301	0,026	6196	0,105	0,013	0,00E+00	0,00E+00	YES
1-erucoyl-GPC (22:1)*	HMDB0010399	Lyso-PC	1102	-0,119	0,029	4783	0,033	0,014	5,62E-06	8,14E-06	YES
1-adrenoyl-GPC (22:4)*	HMDB0010401	Lyso-PC	1329	-0,215	0,027	6173	0,06	0,013	0,00E+00	4,24E-13	YES
1-lignoceroyl-GPC (24:0)	HMDB0010405	Lyso-PC	1193	-0,339	0,029	5345	0,102	0,013	0,00E+00	0,00E+00	YES
1-linolenoyl-GPC (18:3)*	HMDB0010388	Lyso-PC	1332	-0,313	0,026	6197	0,091	0,013	0,00E+00	0,00E+00	YES
1-eicosapentaenoyl-GPC (20:5)*	-	Lyso-PC	1332	0,125	0,025	6199	-0,058	0,013	1,17E-09	1,09E-09	YES
1-eicosenoyl-GPC (20:1)*	HMDB0010391	Lyso-PC	1331	-0,196	0,026	6185	0,057	0,013	5,00E-17	1,01E-12	YES
1-nonadecanoyl-GPC (19:0)	-	Lyso-PC	1245	-0,338	0,027	5834	0,102	0,013	0,00E+00	0,00E+00	YES
1-docosapentaenoyl-GPC (22:5n6)*	-	Lyso-PC	1175	-0,167	0,031	5413	0,047	0,013	2,30E-11	2,10E-10	YES
1-nonadecenoyl-GPC (19:1)*	-	Lyso-PC	1272	-0,242	0,028	5872	0,074	0,013	0,00E+00	0,00E+00	YES
1-palmitoyl-GPE (16:0)	HMDB0011503	Lyso-PE	1331	-0,147	0,027	6184	0,028	0,013	7,35E-09	1,04E-07	YES
1-linoleoyl-GPE (18:2)*	HMDB0011507	Lyso-PE	1332	-0,349	0,026	6195	0,108	0,013	0,00E+00	0,00E+00	YES
1-arachidonoyl-GPE (20:4n6)*	HMDB0011517	Lyso-PE	1331	0,121	0,027	6195	-0,052	0,013	8,64E-09	1,03E-11	YES
2-palmitoyl-GPE (16:0)*	HMDB0011503	Lyso-PE	1327	-0,115	0,025	6174	0,019	0,013	9,50E-06	4,02E-05	YES
2-arachidonoyl-GPE (20:4)*	HMDB0011487	Lyso-PE	1205	0,115	0,027	5304	-0,043	0,014	7,53E-07	1,67E-08	YES

2-linoleoyl-GPE (18:2)*	HMDB0011477	Lyso-PE	1296	-0,297	0,025	5964	0,093	0,013	0,00E+00	0,00E+00	YES
1-docosahexaenoyl-GPE (22:6)*	HMDB11496	Lyso-PE	1332	0,199	0,024	6200	-0,072	0,013	0,00E+00	1,01E-14	YES
2-docosahexaenoyl-GPE (22:6)*	HMDB0011496	Lyso-PE	1199	0,173	0,025	5162	-0,065	0,014	1,15E-13	2,04E-10	YES
1-linolenoyl-GPE (18:3)*	HMDB0011479	Lyso-PE	1177	-0,189	0,028	5523	0,05	0,014	8,97E-14	7,76E-11	YES
**1-palmitoyl-GPI (16:0)**	**HMDB0061695**	**Lyso-PI**	**1321**	**-0,467**	**0,027**	**6124**	**0,151**	**0,012**	**0,00E+00**	**0,00E+00**	YES
1-oleoyl-GPI (18:1)	HMDB0061693	Lyso-PI	1331	-0,421	0,027	6195	0,133	0,012	0,00E+00	0,00E+00	YES
1-linoleoyl-GPI (18:2)*	-	Lyso-PI	1330	-0,442	0,026	6195	0,154	0,012	0,00E+00	0,00E+00	YES
1-palmitoleoyl-GPI (16:1)*	HMDB0061695	Lyso-PI	958	-0,369	0,032	4668	0,101	0,014	0,00E+00	0,00E+00	YES
2-stearoyl-GPI (18:0)*	-	Lyso-PI	1281	-0,255	0,027	5795	0,08	0,013	0,00E+00	0,00E+00	YES
1-palmityl-GPC (O-16:0)	-	Choline-lysoplasmalogen	1330	-0,295	0,026	6177	0,097	0,013	0,00E+00	0,00E+00	YES
1-(1-enyl-palmitoyl)-GPC (P-16:0)*	HMDB0010407	Choline-lysoplasmalogen	1332	-0,31	0,027	6191	0,095	0,013	0,00E+00	0,00E+00	YES
1-(1-enyl-oleoyl)-GPC (P-18:1)*	HMDB0010408	Choline-lysoplasmalogen	1296	-0,268	0,027	6035	0,085	0,013	0,00E+00	0,00E+00	YES
1-(1-enyl-stearoyl)-GPC (P-18:0)*	HMDB0013122	Choline-lysoplasmalogen	1229	-0,316	0,028	5656	0,102	0,013	0,00E+00	0,00E+00	YES
**1-stearyl-GPC (O-18:0)***	**HMDB0011149**	**Choline-lysoplasmalogen**	**1281**	**-0,327**	**0,027**	**5991**	**0,111**	**0,013**	**0,00E+00**	**0,00E+00**	YES
1-(1-enyl-stearoyl)-GPE (P-18:0)*	-	Ethanolamine-lysoplasmalogen	1332	-0,16	0,026	6199	0,053	0,013	1,81E-12	2,82E-08	YES
1-(1-enyl-stearoyl)-2-oleoyl-GPC (P-18:0/18:1)	HMDB0011243	Choline-plasmalogen	1315	-0,422	0,027	6050	0,147	0,012	0,00E+00	0,00E+00	YES
1-(1-enyl-palmitoyl)-2-oleoyl-GPC (P-16:0/18:1)*	HMDB0007996	Choline-plasmalogen	1331	-0,39	0,027	6180	0,133	0,012	0,00E+00	0,00E+00	YES
1-(1-enyl-palmitoyl)-2-docosahexaenoyl-GPC (P-16:0/22:6)*	HMDB0011229	Choline-plasmalogen	1332	-0,173	0,026	6184	0,054	0,013	4,01E-14	3,50E-16	YES
1-(1-enyl-palmitoyl)-2-linoleoyl-GPC (P-16:0/18:2)*	HMDB0011211	Choline-plasmalogen	1330	-0,454	0,028	6179	0,156	0,012	0,00E+00	0,00E+00	YES
1-(1-enyl-stearoyl)-2-docosahexaenoyl-GPC (P-18:0/22:6)*	HMDB0011262	Choline-plasmalogen	1330	-0,168	0,026	6173	0,056	0,013	1,22E-13	1,39E-13	YES
1-palmityl-2-oleoyl-GPC (O-16:0/18:1)*	-	Choline-plasmalogen	1331	-0,359	0,027	6176	0,129	0,012	0,00E+00	0,00E+00	YES
1-(1-enyl-stearoyl)-2-linoleoyl-GPC (P-18:0/18:2)*	HMDB0011244	Choline-plasmalogen	1324	-0,427	0,028	6157	0,148	0,012	0,00E+00	0,00E+00	YES
1-(1-enyl-palmitoyl)-2-palmitoleoyl-GPC (P-16:0/16:1)*	HMDB0011207	Choline-plasmalogen	1324	-0,368	0,029	6140	0,126	0,012	0,00E+00	0,00E+00	YES

1-(1-enyl-palmitoyl)-2-myristoyl-GPC (P-16:0/14:0)*	HMDB0011203	Choline-plasmalogen	1286	-0,407	0,028	6069	0,136	0,012	0,00E+00	0,00E+00	YES
1-(1-enyl-palmitoyl)-2-palmitoyl-GPC (P-16:0/16:0)*	HMDB0011206	Choline-plasmalogen	1331	-0,537	0,027	6179	0,18	0,012	0,00E+00	0,00E+00	YES
**1-palmityl-2-palmitoyl-GPC (O-16:0/16:0)***	**-**	**Choline-plasmalogen**	**1330**	**-0,498**	**0,026**	**6179**	**0,169**	**0,012**	**0,00E+00**	**0,00E+00**	YES
1-(1-enyl-stearoyl)-2-oleoyl-GPE (P-18:0/18:1)	HMDB0011375	Ethanolamine-plasmalogen	1318	-0,24	0,028	6106	0,07	0,013	0,00E+00	0,00E+00	YES
1-(1-enyl-palmitoyl)-2-oleoyl-GPE (P-16:0/18:1)*	HMDB0011342	Ethanolamine-plasmalogen	1325	-0,208	0,027	6143	0,064	0,013	0,00E+00	6,00E-17	YES
**1-(1-enyl-palmitoyl)-2-linoleoyl-GPE (P-16:0/18:2)***	**HMDB0011343**	**Ethanolamine-plasmalogen**	**1327**	**-0,265**	**0,027**	**6157**	**0,092**	**0,013**	**0,00E+00**	**0,00E+00**	YES
1-(1-enyl-stearoyl)-2-linoleoyl-GPE (P-18:0/18:2)*	HMDB0011376	Ethanolamine-plasmalogen	1327	-0,393	0,027	6146	0,132	0,012	0,00E+00	0,00E+00	YES
**Steroids**											
**Dehydroepiandrosterone sulfate (DHEA-S)**	**HMDB0001032**	**Androgenic Steroids**	**1332**	**-0,285**	**0,03**	**6200**	**0,1**	**0,012**	**0,00E+00**	**0,00E+00**	YES
Androstenediol (3beta,17beta) disulfate (1)	HMDB0240313	Androgenic Steroids	1332	-0,113	0,028	6200	0,034	0,012	9,71E-07	3,50E-05	YES
Androstenediol (3alpha, 17alpha) monosulfate (2)	-	Androgenic Steroids	1297	-0,192	0,027	6046	0,071	0,013	1,00E-17	1,52E-07	YES
Androstenediol (3alpha, 17alpha) monosulfate (3)	-	Androgenic Steroids	1330	-0,175	0,029	6195	0,064	0,012	1,57E-15	2,64E-09	YES
Androstenediol (3beta,17beta) monosulfate (1)	-	Androgenic Steroids	1330	-0,235	0,03	6197	0,07	0,012	0,00E+00	1,30E-10	YES
16a-hydroxy DHEA 3-sulfate	HMDB0062544	Androgenic Steroids	1330	-0,104	0,029	6198	0,054	0,012	1,39E-07	4,84E-06	YES
Etiocholanolone glucuronide	-	Androgenic Steroids	1298	-0,150	0,029	6097	0,052	0,012	2,64E-11	2,04E-05	YES
Pregnenediol disulfate (C21H34O8S2)*		Pregnenolone Steroids	1332	-0,247	0,028	6200	0,097	0,012	0,00E+00	0,00E+00	YES
21-hydroxypregnenolone disulfate	-	Pregnenolone Steroids	1324	-0,228	0,028	6184	0,084	0,012	0,00E+00	1,22E-15	YES
**Pregnenediol sulfate (C21H34O5S)***	**HMDB0000774**	**Pregnenolone Steroids**	**1332**	**-0,350**	**0,027**	**6200**	**0,126**	**0,012**	**0,00E+00**	**0,00E+00**	YES
17alpha-hydroxypregnenolone 3-sulfate	HMDB0000416	Pregnenolone Steroids	832	-0,255	0,034	4110	0,089	0,015	0,00E+00	1,69E-12	YES
Pregnenolone sulfate	HMDB0000774	Pregnenolone Steroids	1295	-0,271	0,027	6076	0,098	0,013	0,00E+00	0,00E+00	YES

17alpha-hydroxypregnanolone glucuronide	-	Pregnenolone Steroids	635	-0,157	0,039	2803	0,056	0,019	1,12E-06	2,13E-06	YES
Pregnenetriol sulfate*	-	Pregnenolone Steroids	1332	-0,337	0,029	6200	0,132	0,012	0,00E+00	0,00E+00	YES
Pregnenetriol disulfate*	-	Pregnenolone Steroids	1332	-0,203	0,029	6200	0,086	0,012	0,00E+00	0,00E+00	YES
5alpha-pregnan-3beta,20beta-diol monosulfate (1)	HMDB0240580	Progestin Steroids	1212	-0,145	0,029	5569	0,052	0,013	4,39E-10	7,18E-07	YES
**Cholesterol**	**HMDB0000067**	**Sterol**	**1332**	**-0,658**	**0,026**	**6200**	**0,208**	**0,012**	**0,00E+00**	**0,00E+00**	**Yes**
7-alpha-hydroxy-3-oxo-4-cholestenoate (7-Hoca)	HMDB0012458	Sterol	1332	-0,118	0,027	6200	0,023	0,013	3,67E-06	5,17E-13	YES
4-cholesten-3-one	HMDB0000921	Sterol	920	-0,255	0,035	4284	0,07	0,015	0,00E+00	0,00E+00	YES
3beta-hydroxy-5-cholestenoate	-	Sterol	1332	-0,138	0,027	6199	0,039	0,013	4,45E-09	1,61E-06	YES
**Fatty acids**											
Laurate (12:0)	HMDB0000638	Medium Chain FA	1332	-0,148	0,027	6200	0,039	0,013	6,63E-10	6,50E-10	YES
**Caprate (10:0)**	**HMDB0000511**	**Medium Chain FA**	**1332**	**-0,167**	**0,027**	**6200**	**0,044**	**0,013**	**3,06E-12**	**3,25E-14**	YES
5-dodecenoate (12:1n7)	HMDB0000529	Medium Chain FA	1332	-0,1	0,027	6200	0,024	0,013	4,11E-05	4,15E-07	YES
Myristoleate (14:1n5)	HMDB0002000	Long Chain MUFA	1332	-0,134	0,027	6200	0,034	0,013	2,13E-08	2,46E-12	YES
10-nonadecenoate (19:1n9)	HMDB0013622	Long Chain MUFA	1332	-0,105	0,027	6200	0,03	0,013	7,39E-06	2,97E-11	YES
Palmitate (16:0)	HMDB0000220	Long Chain Saturated FA	1332	-0,103	0,028	6200	0,024	0,013	2,36E-05	9,96E-10	YES
Stearate (18:0)	HMDB0000827	Long Chain Saturated FA	1332	-0,112	0,027	6200	0,028	0,013	3,72E-06	2,46E-09	YES
**Myristate (14:0)**	**HMDB0000806**	**Long Chain Saturated FA**	**1332**	**-0,152**	**0,027**	**6200**	**0,042**	**0,013**	**1,25E-10**	**4,40E-16**	YES
Nonadecanoate (19:0)	HMDB0000772	Long Chain Saturated FA	1332	-0,122	0,028	6199	0,042	0,013	5,54E-08	9,84E-10	YES
Arachidate (20:0)	HMDB0002212	Long Chain Saturated FA	1332	-0,163	0,027	6200	0,047	0,013	3,91E-12	7,86E-10	YES
Pentadecanoate (15:0)	HMDB0000826	Long Chain Saturated FA	1332	-0,104	0,027	6200	0,033	0,013	6,29E-06	7,38E-10	YES
Picolinoylglycine	HMDB0059766	FA Metabolism (Acyl Glycine)	1316	0,175	0,027	6016	-0,071	0,013	4,40E-16	1,37E-06	YES
Malonylcarnitine	HMDB0002095	FA Synthesis	1125	0,112	0,03	5009	-0,053	0,014	4,29E-07	2,43E-05	YES
**Pristanate**	**HMDB0000795**	**FA, Branched**	**1285**	**-0,217**	**0,028**	**6024**	**0,057**	**0,013**	**0,00E+00**	**0,00E+00**	YES
(16 or 17)-methylstearate (a19:0 or i19:0)	HMDB0037397	FA, Branched	1331	-0,117	0,027	6196	0,036	0,013	3,81E-07	8,74E-12	YES
(14 or 15)-methylpalmitate (a17:0 or i17:0)	HMDB0061859	FA, Branched	1319	-0,15	0,028	6151	0,044	0,013	1,61E-10	7,21E-15	YES

Cis-3,4-methyleneheptanoate	-	FA, Branched	1325	0,18	0,026	6180	-0,052	0,013	1,41E-14	9,10E-16	YES
3-carboxy-4-methyl-5-propyl-2-furanpropanoate (CMPF)	HMDB0061112	Furanoid FA	1332	0,175	0,025	6200	-0,058	0,013	1,48E-14	6,44E-06	YES
**Hydroxy fatty acids**											YES
2S,3R-dihydroxybutyrate	HMDB0002453	FA, Dihydroxy	1332	0,11	0,028	6197	-0,035	0,013	1,56E-06	3,28E-07	YES
2R,3R-dihydroxybutyrate	HMDB0000498	FA, Dihydroxy	1332	0,159	0,027	6199	-0,056	0,013	6,20E-13	4,91E-09	YES
2-hydroxystearate	HMDB0062549	FA, Monohydroxy	1332	-0,215	0,027	6200	0,062	0,013	0,00E+00	0,00E+00	YES
3-hydroxylaurate	HMDB0000387	FA, Monohydroxy	1332	-0,11	0,027	6198	0,023	0,013	1,08E-05	4,24E-09	YES
**2-hydroxypalmitate**	**HMDB0031057**	**FA, Monohydroxy**	**1332**	**-0,222**	**0,027**	**6200**	**0,068**	**0,013**	**0,00E+00**	**0,00E+00**	YES
9-hydroxystearate	HMDB0061661	FA, Monohydroxy	1206	-0,132	0,027	5356	0,032	0,014	2,41E-07	7,97E-10	YES
2-hydroxybehenate	HMDB0061660	FA, Monohydroxy	1315	-0,122	0,026	6026	0,042	0,013	7,74E-08	5,20E-09	YES
**Acylcarnitines**											YES
Adipoylcarnitine (C6-DC)	HMDB0061677	Acyl Carnitine, Dicarboxylate	1323	0,176	0,029	6158	-0,053	0,012	2,19E-14	9,32E-09	YES
Stearoylcarnitine (C18)	HMDB0000848	Acyl Carnitine, Long Chain Saturated	1332	-0,221	0,027	6199	0,061	0,013	0,00E+00	0,00E+00	YES
Margaroylcarnitine (C17)*	HMDB0006210	Acyl Carnitine, Long Chain Saturated	1272	-0,141	0,029	5690	0,04	0,013	5,13E-09	4,69E-13	YES
Behenoylcarnitine (C22)*	HMDB0062468	Acyl Carnitine, Long Chain Saturated	993	-0,199	0,031	4117	0,068	0,016	3,58E-14	4,37E-13	YES
Arachidoylcarnitine (C20)*	HMDB0006460	Acyl Carnitine, Long Chain Saturated	1130	-0,277	0,029	4630	0,092	0,015	0,00E+00	0,00E+00	YES
Lignoceroylcarnitine (C24)*	-	Acyl Carnitine, Long Chain Saturated	1289	-0,243	0,027	5895	0,074	0,013	0,00E+00	0,00E+00	YES
**Cerotoylcarnitine (C26)***	**HMDB0006347**	**Acyl Carnitine, Long Chain Saturated**	**1318**	**-0,33**	**0,028**	**6103**	**0,099**	**0,013**	**0,00E+00**	**0,00E+00**	YES
Hexanoylcarnitine (C6)	HMDB0000756	Acyl Carnitine, Medium Chain	1332	0,148	0,028	6200	-0,057	0,012	6,99E-12	7,56E-06	YES
Octanoylcarnitine (C8)	HMDB0000791	Acyl Carnitine, Medium Chain	1332	0,129	0,029	6200	-0,048	0,012	3,75E-09	9,11E-07	YES
**Cis-3,4-methyleneheptanoylcarnitine**	**-**	**Acyl Carnitine, Medium Chain**	**1332**	**0,199**	**0,026**	**6199**	**-0,065**	**0,013**	**0,00E+00**	**6,84E-15**	YES
(S)-3-hydroxybutyrylcarnitine	HMDB0013127	Acyl Carnitine, Short Chain	1313	0,192	0,027	6061	-0,057	0,013	2,80E-16	3,52E-10	YES

Cis-4-decenoylcarnitine (C10:1)	HMDB0013205	Acyl Carnitine, MU	1332	0,186	0,028	6200	-0,066	0,012	4,00E-17	5,52E-13	YES
Ximenoylcarnitine (C26:1)*	-	Acyl Carnitine, MU	1293	-0,273	0,028	5936	0,08	0,013	0,00E+00	0,00E+00	YES
Nervonoylcarnitine (C24:1)*	-	Acyl Carnitine, MU	1179	-0,128	0,029	5008	0,035	0,014	4,41E-07	3,22E-09	YES
Linolenoylcarnitine (C18:3)*	-	Acyl Carnitine, PU	1294	0,109	0,028	5848	-0,042	0,013	7,82E-07	4,38E-05	YES
Arachidonoylcarnitine (C20:4)	HMDB0006455	Acyl Carnitine, PU	1277	0,2	0,028	5611	-0,089	0,013	0,00E+00	1,00E-17	YES
Dihomo-linolenoylcarnitine (C20:3n3 or 6)*	-	Acyl Carnitine, PU	1265	0,1	0,027	5672	-0,049	0,013	1,18E-06	3,14E-05	YES
**Docosahexaenoylcarnitine (C22:6)***	**-**	**Acyl Carnitine, PU**	**1200**	**0,22**	**0,028**	**5006**	**-0,087**	**0,014**	**0,00E+00**	**1,03E-14**	YES
Docosapentaenoylcarnitine (C22:5n3)*	-	Acyl Carnitine, PU	1076	0,147	0,03	4361	-0,075	0,015	4,65E-11	1,23E-07	YES
**Acylcholines**											YES
Palmitoylcholine	HMDB0240592	Acyl Choline	1332	-0,15	0,027	6199	0,049	0,013	2,72E-11	1,16E-07	YES
Oleoylcholine	HMDB0240596	Acyl Choline	1332	-0,14	0,027	6190	0,043	0,013	1,08E-09	4,69E-06	YES
Palmitoloelycholine	-	Acyl Choline	951	-0,156	0,031	4759	0,045	0,014	1,20E-08	1,73E-07	YES
**Linoleoylcholine***	**HMDB0013213**	**Acyl Choline**	**1331**	**-0,238**	**0,027**	**6193**	**0,084**	**0,012**	**0,00E+00**	**0,00E+00**	YES
**Dicarboxylic acids**											
Tetradecanedioate (C14-DC)	HMDB0000872	FA, Dicarboxylate	1332	-0,101	0,027	6197	0,033	0,013	9,28E-06	4,30E-09	YES
**Sphingolipids**											
Palmitoyl-sphingosine-phosphoethanolamine (d18:1/16:0)	-	Ceramide PEs	1312	-0,508	0,027	6040	0,176	0,012	0,00E+00	0,00E+00	YES
**N-palmitoyl-sphingosine (d18:1/16:0)**	**HMDB0004949**	**Ceramides**	**1332**	**-0,342**	**0,027**	**6200**	**0,106**	**0,012**	**0,00E+00**	**0,00E+00**	YES
N-palmitoyl-sphingadienine (d18:2/16:0)*	-	Ceramides	1183	-0,136	0,029	5296	0,051	0,014	5,72E-09	3,48E-10	YES
N-behenoyl-sphingadienine (d18:2/22:0)*	-	Ceramides	1032	-0,128	0,03	3996	0,038	0,016	1,99E-06	2,29E-06	YES
Ceramide (d16:1/24:1, d18:1/22:1)*	-	Ceramides	1065	-0,135	0,03	4148	0,032	0,016	1,28E-06	5,00E-08	YES
N-palmitoyl-heptadecasphingosine (d17:1/16:0)*	-	Ceramides	1038	-0,27	0,032	4546	0,093	0,015	0,00E+00	0,00E+00	YES
Ceramide (d18:1/14:0, d16:1/16:0)*	-	Ceramides	1133	-0,162	0,029	5168	0,054	0,014	5,40E-11	1,94E-13	YES
Ceramide (d18:2/24:1, d18:1/24:2)*	-	Ceramides	1079	-0,189	0,029	4203	0,053	0,015	8,63E-13	1,66E-15	YES
N-palmitoyl-sphinganine (d18:0/16:0)	HMDB0011760	Dihydroceramides	1181	-0,267	0,031	5036	0,089	0,014	0,00E+00	0,00E+00	YES
Palmitoyl dihydrosphingomyelin (d18:0/16:0)*	HMDB0010168	Dihydrosphingomyelins	1331	-0,452	0,027	6179	0,162	0,012	0,00E+00	0,00E+00	YES

Behenoyl dihydrosphingomyelin (d18:0/22:0)*	HMDB0012091	Dihydrosphingomyelins	1177	-0,119	0,028	4956	0,057	0,014	3,57E-08	9,91E-08	YES
**Myristoyl dihydrosphingomyelin (d18:0/14:0)***	**HMDB0012085**	**Dihydrosphingomyelins**	**1326**	**-0,336**	**0,027**	**6134**	**0,12**	**0,012**	**0,00E+00**	**0,00E+00**	YES
Sphingomyelin (d18:0/20:0, d16:0/22:0)*	-	Dihydrosphingomyelins	1325	-0,091	0,026	6094	0,043	0,013	9,14E-06	3,26E-06	YES
Glycosyl-N-stearoyl-sphingosine (d18:1/18:0)	-	Hexosylceramides	1242	-0,441	0,028	5521	0,156	0,013	0,00E+00	0,00E+00	YES
Glycosyl-N-palmitoyl-sphingosine (d18:1/16:0)	-	Hexosylceramides	1332	-0,668	0,027	6200	0,217	0,011	0,00E+00	0,00E+00	YES
Glycosyl-N-behenoyl-sphingadienine (d18:2/22:0)*	-	Hexosylceramides	1075	-0,542	0,031	4335	0,202	0,014	0,00E+00	0,00E+00	YES
Glycosyl ceramide (d16:1/24:1, d18:1/22:1)*	-	Hexosylceramides	627	-0,35	0,04	2533	0,121	0,019	0,00E+00	0,00E+00	YES
Glycosyl ceramide (d18:2/24:1, d18:1/24:2)*	-	Hexosylceramides	1097	-0,493	0,031	4369	0,185	0,014	0,00E+00	0,00E+00	YES
Glycosyl-N-(2-hydroxynervonoyl)-sphingosine (d18:1/24:1(2OH))*	-	Hexosylceramides	1057	-0,128	0,031	4174	0,03	0,016	4,51E-06	0,00E+00	YES
**Glycosyl ceramide (d18:1/20:0, d16:1/22:0)***	**-**	**Hexosylceramides**	**1098**	**-0,563**	**0,03**	**4368**	**0,209**	**0,014**	**0,00E+00**	**0,00E+00**	YES
Lactosyl-N-palmitoyl-sphingosine (d18:1/16:0)	-	Lactosylceramides	1332	-0,506	0,026	6200	0,172	0,012	0,00E+00	0,00E+00	YES
Lactosyl-N-nervonoyl-sphingosine (d18:1/24:1)*	-	Lactosylceramides	1102	-0,543	0,03	4371	0,211	0,014	0,00E+00	0,00E+00	YES
Lactosyl-N-behenoyl-sphingosine (d18:1/22:0)*	HMDB0011594	Lactosylceramides	916	-0,306	0,033	3850	0,128	0,016	0,00E+00	0,00E+00	YES
Sphingomyelin (d18:1/18:1, d18:2/18:0)	HMDB0012101	Sphingomyelins	1332	0,115	0,026	6193	-0,033	0,013	1,05E-06	2,67E-05	YES
**Palmitoyl sphingomyelin (d18:1/16:0)**	**HMDB0010169**	**Sphingomyelins**	**1331**	**-0,526**	**0,025**	**6192**	**0,181**	**0,012**	**0,00E+00**	**0,00E+00**	YES
Sphingomyelin (d18:1/14:0, d16:1/16:0)*	HMDB0012097	Sphingomyelins	1332	-0,433	0,027	6200	0,144	0,012	0,00E+00	0,00E+00	YES
Sphingomyelin (d18:2/16:0, d18:1/16:1)*	-	Sphingomyelins	1332	-0,349	0,024	6194	0,12	0,013	0,00E+00	0,00E+00	YES

Sphingomyelin (d18:2/14:0, d18:1/14:1)*	-	Sphingomyelins	1331	-0,277	0,027	6177	0,085	0,013	0,00E+00	0,00E+00	YES
Sphingomyelin (d18:1/24:1, d18:2/24:0)*	HMDB0012107	Sphingomyelins	1321	-0,282	0,023	6104	0,098	0,013	0,00E+00	0,00E+00	YES
Sphingomyelin (d18:1/20:0, d16:1/22:0)*	HMDB0012102	Sphingomyelins	1331	-0,133	0,025	6178	0,049	0,013	1,98E-09	3,55E-08	YES
Behenoyl sphingomyelin (d18:1/22:0)*	HMDB0012103	Sphingomyelins	1317	-0,364	0,027	6050	0,132	0,012	0,00E+00	0,00E+00	YES
Sphingomyelin (d18:1/22:1, d18:2/22:0, d16:1/24:1)*	HMDB0012104	Sphingomyelins	1330	-0,294	0,031	6177	0,098	0,012	0,00E+00	0,00E+00	YES
Sphingomyelin (d18:1/22:2, d18:2/22:1, d16:1/24:2)*	-	Sphingomyelins	1331	-0,277	0,026	6186	0,106	0,013	0,00E+00	0,00E+00	YES
Lignoceroyl sphingomyelin (d18:1/24:0)	-	Sphingomyelins	1102	-0,44	0,03	4372	0,171	0,014	0,00E+00	0,00E+00	YES
Sphingomyelin (d17:1/16:0, d18:1/15:0, d16:1/17:0)*	-	Sphingomyelins	1331	-0,423	0,026	6192	0,15	0,012	0,00E+00	0,00E+00	YES
Sphingomyelin (d18:1/17:0, d17:1/18:0, d19:1/16:0)	-	Sphingomyelins	1331	-0,198	0,025	6189	0,081	0,013	0,00E+00	0,00E+00	YES
Tricosanoyl sphingomyelin (d18:1/23:0)*	HMDB0012105	Sphingomyelins	1232	-0,308	0,024	5358	0,121	0,014	0,00E+00	0,00E+00	YES
Sphingomyelin (d18:2/23:0, d18:1/23:1, d17:1/24:1)*	-	Sphingomyelins	1330	-0,376	0,027	6176	0,131	0,012	0,00E+00	0,00E+00	YES
Sphingomyelin (d18:2/24:1, d18:1/24:2)*	-	Sphingomyelins	1331	-0,28	0,024	6182	0,096	0,013	0,00E+00	0,00E+00	YES
Sphingomyelin (d18:1/21:0, d17:1/22:0, d16:1/23:0)*	-	Sphingomyelins	1328	-0,255	0,027	6169	0,087	0,013	0,00E+00	0,00E+00	YES
Sphingomyelin (d18:2/18:1)*	HMDB0001348	Sphingomyelins	1329	-0,105	0,026	6170	0,058	0,013	4,36E-08	8,65E-07	YES
Sphingomyelin (d18:2/24:2)*	-	Sphingomyelins	1330	-0,274	0,028	6174	0,1	0,012	0,00E+00	0,00E+00	YES
Sphingomyelin (d18:2/21:0, d16:2/23:0)*	-	Sphingomyelins	1328	-0,163	0,027	6152	0,058	0,013	2,76E-13	9,00E-17	YES
Sphingomyelin (d18:2/23:1)*	-	Sphingomyelins	1326	-0,338	0,028	6151	0,114	0,012	0,00E+00	0,00E+00	YES
Sphingomyelin (d18:1/25:0, d19:0/24:1, d20:1/23:0, d19:1/24:0)*	-	Sphingomyelins	1102	-0,18	0,03	4371	0,081	0,015	4,13E-15	8,69E-12	YES
Sphingomyelin (d17:2/16:0, d18:2/15:0)*	-	Sphingomyelins	1300	-0,253	0,028	5960	0,087	0,013	0,00E+00	0,00E+00	YES

Sphingomyelin (d17:1/14:0, d16:1/15:0)*	-	Sphingomyelins	1318	-0,338	0,027	6109	0,113	0,012	0,00E+00	0,00E+00	YES
Hydroxypalmitoyl sphingomyelin (d18:1/16:0(OH))**	-	Sphingomyelins	1331	-0,494	0,026	6178	0,17	0,012	0,00E+00	0,00E+00	YES
Sphingosine 1-phosphate	HMDB0000277	Sphingosines	1332	-0,126	0,027	6199	0,042	0,013	2,58E-08	1,68E-09	YES
**Bile acids**											
**Deoxycholate**	**HMDB0000626**	**Secondary BA Metabolism**	**1169**	**-0,121**	**0,029**	**5542**	**0,041**	**0,013**	**3,72E-07**	**1,28E-10**	**No(69)**
Glycodeoxycholate	HMDB0000631	Secondary BA Metabolism	960	-0,114	0,032	4664	0,041	0,015	1,15E-05	1,99E-10	YES
Glycocholenate sulfate*	-	Secondary BA Metabolism	1332	-0,098	0,029	6199	0,047	0,012	1,53E-06	1,37E-09	YES
3b-hydroxy-5-cholenoic acid	HMDB0000308	Secondary BA Metabolism	1043	-0,155	0,033	4616	0,047	0,014	3,77E-09	8,32E-08	YES
Glycodeoxycholate 3-sulfate	-	Secondary BA Metabolism	1237	-0,088	0,029	5848	0,042	0,013	2,70E-05	6,40E-06	YES
**Mevalonate metabolism**											
3-hydroxy-3-methylglutarate	HMDB0000355	Mevalonate Metabolism	1332	0,676	0,027	6187	-0,233	0,011	0,00E+00	0,00E+00	YES
**N-acyl-amines**											
**N-stearoyltaurine**	**-**	**-**	**1128**	**-0,15**	**0,029**	**4748**	**0,041**	**0,015**	**9,56E-09**	**2,55E-12**	YES
N-stearoylserine*	-	-	1101	-0,114	0,029	4797	0,044	0,015	2,55E-06	1,88E-06	YES
**Cofactors and Vitamins**											
N1-Methyl-2-pyridone-5-carboxamide	HMDB0004193	Nicotinate and Nicotinamide Metabolism	1332	0,146	0,028	6200	-0,035	0,013	1,72E-09	1,47E-06	YES
**Alpha-tocopherol**	**HMDB0001893**	**Tocopherol Metabolism**	**1332**	**-0,151**	**0,027**	**6199**	**0,039**	**0,013**	**2,09E-10**	**1,39E-09**	**No(30)**
**Energy metabolism**											
**Succinylcarnitine (C4-DC)**	**HMDB0061717**	**TCA Cycle**	**1329**	**0,132**	**0,028**	**6158**	**-0,049**	**0,013**	**1,53E-09**	**3,46E-05**	YES
**Xenobiotics**											
**2-hydroxyhippurate (salicylurate)**	**HMDB0000840**	**Benzoate Metabolism**	**1124**	**0,365**	**0,035**	**4524**	**-0,134**	**0,013**	**0,00E+00**	**0,00E+00**	YES
Guaiacol sulfate	HMDB0060013	Benzoate Metabolism	1330	0,137	0,028	6199	-0,047	0,013	1,16E-09	2,08E-05	YES
Sulfate*	HMDB01448	Chemical	1332	0,214	0,029	6200	-0,065	0,012	0,00E+00	2,91E-12	YES
2,4-di-tert-butylphenol	-	Chemical	1186	0,106	0,029	6195	0,036	0,013	1,79E-08	5,25E-08	YES
Salicyluric glucuronide*	HMDB0240252	Drug - Analgesics, Anesthetics	611	0,167	0,039	1028	-0,127	0,032	1,47E-08	5,35E-07	YES

Salicylate	HMDB0001895	Drug - Topical Agents	1296	0,358	0,034	5854	-0,117	0,012	0,00E+00	0,00E+00	YES
Phytanate	HMDB0000801	Food Component/Plant	1332	-0,211	0,026	6200	0,068	0,013	0,00E+00	0,00E+00	YES
Erythritol	HMDB0002994	Food Component/Plant	1330	0,178	0,028	6193	-0,049	0,012	2,91E-14	2,93E-06	YES
Homostachydrine*	HMDB0033433	Food Component/Plant	1328	0,175	0,027	6123	-0,042	0,013	1,02E-12	3,23E-11	YES
5-hydroxymethyl-2-furoylcarnitine*	-	Food Component/Plant	980	0,175	0,033	4204	-0,07	0,015	2,90E-12	9,35E-08	YES
**Other metabolites**											
Glycine conjugate of C10H14O2 (1)*	-	Partially Characterized	1285	0,155	0,027	5826	-0,055	0,013	8,14E-12	9,48E-09	YES
Glutamine conjugate of C6H10O2 (1)*	-	Partially Characterized	1302	-0,08	0,028	6014	0,044	0,013	4,01E-05	7,38E-09	YES
Metabolonic lactone sulfate	-	Partially Characterized	1319	-0,12	0,028	6149	0,038	0,013	1,71E-07	1,34E-15	YES

*Comparison with ANOVA, statistically significant P <4,5E-05 **P-value adjusted for age, BMI, fasting glucose and smoking. P-value <1,00E-17=0,00E+00. Only statistically significant metabolites in both analysis (ANOVA and ANCOVA) are shown. Most significant metabolites are in bold.

Abbreviations: BCAA, branched chain amino acid; FA, fatty acid; GPC, glycerophosphatidylcholine; GPE, glycerophosphatidylethanolamine; GPI, glycerophosphatidylinositol; Lyso-PC, lysophosphatidylcholine; Lyso-PE, lysophosphatidylethanolamine; PC, phosphatidylcholine; PE, phosphatidylethanolamines; PI, phosphatidylinositol; MU, monounsaturated; PU, polyunsaturated; TCA, tricarboxylic acid cycle.
